# Rupture of equine pericardial aortic-root patch after aortic valve replacement with aortic annulus enlargement: a case report

**DOI:** 10.1186/1749-8090-9-109

**Published:** 2014-06-19

**Authors:** Akimasa Morisaki, Yasuyuki Kato, Manabu Motoki, Yosuke Takahashi, Shinsuke Nishimura, Toshihiko Shibata

**Affiliations:** 1 Department of Cardiovascular Surgery, Osaka City General Hospital, 2-13-22 Miyakojima-hondori, Miyakojima-ku, Osaka 534-0021, Japan

**Keywords:** Aortic annulus enlargement, Equine pericardial patch, Double-layered patch repair, Pericardial patch rupture, Pseudoaneurysm

## Abstract

There are no previous reports of rupture of a heterologous pericardial patch after aortic annulus enlargement. Our patient, a 72-year-old Japanese female, presented with congestive heart failure resulting from heart compression from pseudoaneurysm formation in the aortic root. At 57 years of age the patient had undergone replacement of the ascending aorta for Stanford type A acute aortic dissection. At 66 years of age she had undergone aortic valve replacement with a mechanical valve, accompanied by enlargement of the aortic annulus using an equine pericardial patch, for severe aortic valve stenosis with a narrow aortic annulus. Equine pericardial patch was used in the aortic annulus enlargement to form the aortic root from the ascending aortic vascular prosthesis to the non-coronary cusp of the aortic valve. We performed repeat median sternotomy under cardiopulmonary bypass with moderate hypothermia. The ascending aorta was balloon-occluded because of dense adhesions around the superior vena cava and ascending aorta due to the pseudoaneurysm. A tear in the equine pericardial patch was noted at the aortic root. The patient underwent pseudoaneurysm excision and repair of the aortic root using a double-layered, Hemashield-reinforced bovine pericardial patch. Routine follow-up with computed tomography should be performed for early detection of complications from a heterologous pericardial patch.

## Background

Aortic annulus enlargement using a heterologous pericardial patch is performed in patients with a narrow aortic annulus to prevent patient-prosthesis mismatch [1-3]. Rupture of a heterologous pericardial patch after aortic annulus enlargement has not previously been reported. We present a rare case of rupture of an equine pericardial patch after aortic valve replacement with aortic annulus enlargement.

## Case presentation

### History

A 72-year-old Japanese woman was referred to our institution for treatment of congestive heart failure with dyspnea on exertion. At 57 years of age she had experienced Stanford type A acute aortic dissection and underwent replacement of the ascending aorta under deep hypothermia and retrograde cerebral perfusion using a 30-mm vascular prosthesis without malperfusion. At 66 years of age she was diagnosed with severe aortic valve stenosis with a narrow aortic annulus and underwent aortic valve replacement with a mechanical valve (ATS Open Pivot® AP™ 18 mm; ATS Medical Inc., Minneapolis, MN, USA) accompanied by enlargement of the aortic annulus (Nicks procedure [4]). To enlarge the aortic annulus, a glutaraldehyde-fixed equine pericardial patch was used to form the aortic root from the ascending aortic vascular prosthesis to the non-coronary cusp of the aortic valve. The patient had received warfarin therapy for atrial fibrillation after aortic valve replacement. Six years after the aortic valve replacement with aortic annulus enlargement, she presented with congestive heart failure.

### Imaging

**Figure 1:**
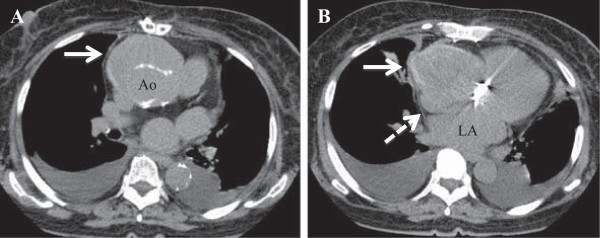
**Computed tomography. (A)** The pseudoaneurysm (solid arrow) anterior to the aortic root and right atrium. **(A, B)** Compression of the superior vena cava and right atrium (dashed arrow). Ao: ascending aortic artery, LA: left atrium.

**Figure 2:**
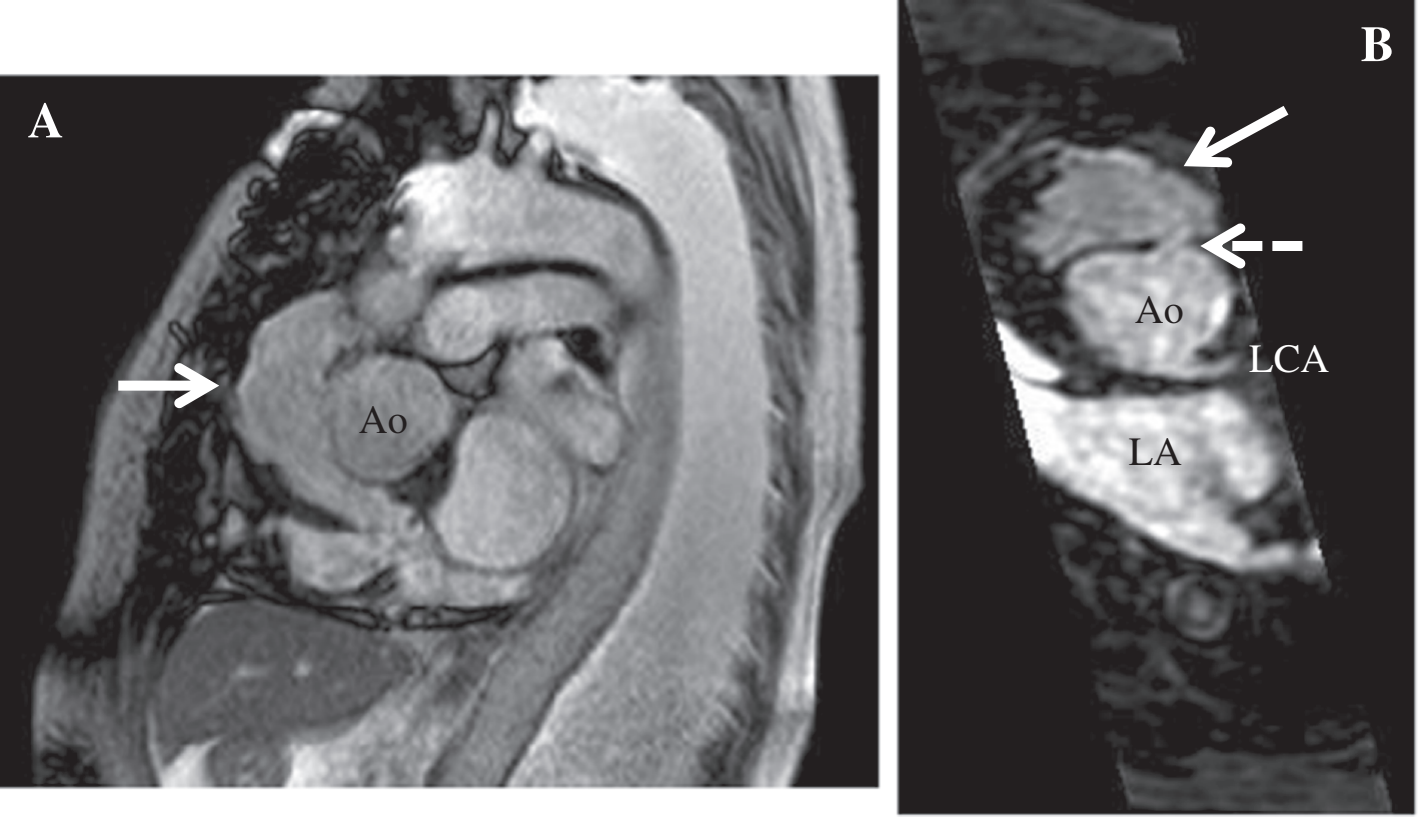
**Magnetic resonance angiography. (A)** Sagittal view revealing the pseudoaneurysm (solid arrow). **(B)** Axial view showing the connection (dashed arrow) between the aortic root to the pseudoaneurysm. Ao: ascending aortic artery, LA: left atrium, LCA: left coronary artery.

Chest x-ray showed cardiomegaly with pleural effusion and pulmonary congestion. Computed tomography (CT) revealed a huge mass consistent with pseudoaneurysm in front of the right atrium and aortic root (Figure 1). Magnetic resonance angiography showed a pseudoaneurysm with blood flow from the aortic root near the sinotubular junction (Figure 2). Echocardiography revealed a pseudoaneurysm with continuous flow from the aortic root, moderate tricuspid regurgitation, and moderate mitral regurgitation. Peak velocity in the aortic mechanical valve was 2.7 m/sec (peak pressure gradient: 27 mmHg), with normal ejection fraction and no left ventricular dilatation. No inflammatory or infectious response was noted.

These findings suggested that the patient’s congestive heart failure was caused by cardiac compression from the pseudoaneurysm.

### Operative technique

**Figure 3:**
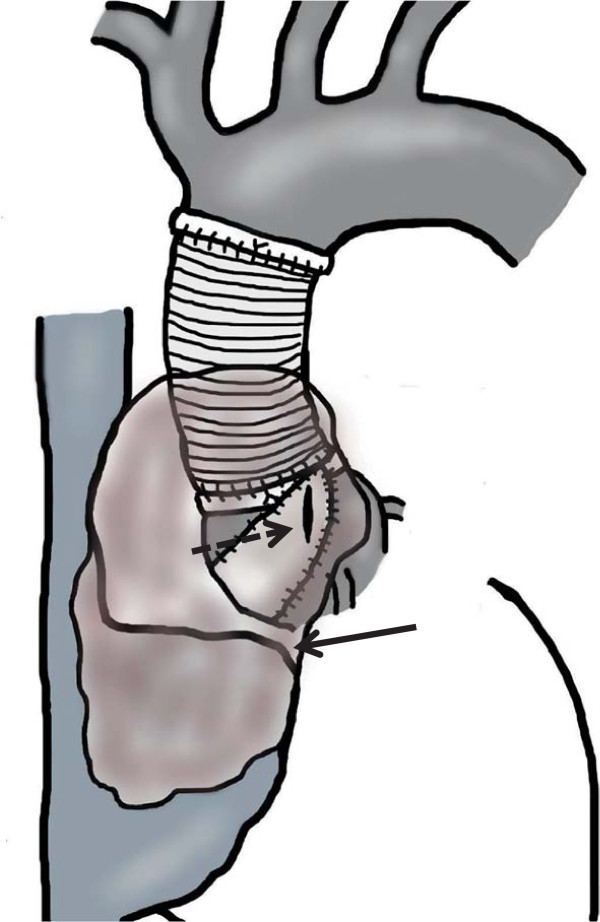
**Intraoperative image.** Dashed arrow showing the laceration in the equine pericardial patch; solid arrow showing the pseudoaneurysm.

Repeat median sternotomy risked cardiac injury or rupture of the pseudoaneurysm. Therefore, we attempted median sternotomy under cardiopulmonary bypass with moderate hypothermia. Additionally, an aortic occlusion balloon was inserted into the ascending aorta through the right femoral artery to prevent bleeding from the pseudoaneurysm. After systemic heparinization, cardiopulmonary bypass was established with an arterial cannula to the left femoral and right axillary arteries and a venous cannula to the right atrium through the left femoral vein. We then performed a repeat median sternotomy with the patient under moderate hypothermia of 28°C. Because of dense adhesions around the superior vena cava and ascending aorta caused by the pseudoaneurysm, lysis of adhesions and clamping of the ascending aorta were not possible. After circulatory arrest, we excised the pseudoaneurysm. We identified a longitudinal tear in the equine pericardial patch near the ascending aortic vascular prosthesis and an intact suture line (Figure 3). The ascending aorta was balloon-occluded and circulation was quickly reestablished. Excision of the pseudoaneurysm with mural thrombus created a defect in the right atrial wall. We removed the equine pericardial patch around the tear and performed patch repair of the aortic root using a double-layered patch consisting of an inner bovine pericardial patch layer and an outer Hemashield patch layer (Hemashield Double Velour Fabric; MAQUET Holding B.V. & Co. KG, Rastatt, Germany) with a felt-reinforced suture line. We then performed tricuspid annuloplasty and repair of the right atrial wall using an equine pericardial patch.

### Postoperative course and follow-up

After the patient was weaned uneventfully from cardiopulmonary bypass, transesophageal echocardiography showed an improvement in mitral valve regurgitation from moderate to mild. The patient was extubated 20 hours after surgery and discharged on postoperative day 20 with improving congestive heart failure. A year after the operation, no pseudoaneurysm-related change was detected.

## Discussion

Enlargement of the aortic annulus using a heterologous pericardial patch is performed extensively in patients with a narrow aortic annulus and has become a simple and reproducible technique for many cardiac surgeons [2]. In a long-term follow-up survey, Celiento et al. reported that in patients with a small aortic annulus, enlargement of the aortic annulus is a safe and effective procedure that does not cause late aneurysm formation or dilatation of the aortic root [3]. Although it is possible for an aneurysm to form in a heterologous pericardial patch and go undetected, there have been no reports of rupture and late aneurysm formation after heterologous pericardial patch repair of aortic annulus enlargement in adults. However, a few cases of aneurysm formation after bovine pericardial patch repair have been reported in children [5,6]. A patient who underwent a Norwood procedure with Sano right ventricle-to-pulmonary artery shunt for hypoplastic left heart syndrome was reported to develop a pseudoaneurysm in the proximal portion of the Sano anastomosis caused by degeneration of the bovine pericardium [5]. Moreover, a neoaortic aneurysm constructed with native great vessel and bovine pericardial patch after third-stage palliation for hypoplastic left heart has been reported [6]. Histopathological changes evident in disruptions of elastic laminae suggest an inherent defect in the patch material rather than in the method of fixation or surgical technique. In vascular surgery, there have been sporadic reports of pseudoaneurysm formation of unclear etiology with bovine pericardial patches [7]. Therefore, it may be necessary to perform periodic CT scans to detect pseudoaneurysm-related changes.

The bovine glutaraldehyde-fixed pericardial patch may be responsible for the low rate of pseudoaneurysm formation; anticalcification technology and acellular pure collagen may provide host-cell migration and proliferation, accelerated endothelialization, and tissue regeneration [7]. However, in our case, the tear in the equine pericardial patch with thinning developed adjacent to the ascending aortic vascular prosthesis. This may indicate that a frictional force on the pericardial patch between the vascular prosthesis and the suture line led to degenerative changes. Considering this possibility, we used a double-layered patch consisting of a bovine pericardial patch reinforced with a Hemashield patch to improve the strength and durability of the repair.

## Conclusion

This was a rare case of rupture of an equine pericardial patch after aortic annulus enlargement that was successfully treated using a double-layered bovine pericardial/Hemashield patch. If a heterologous pericardial patch is used in this procedure, a routine follow-up CT may be necessary for early detection of complications of the patch.

## Consent

Written informed consent was obtained from the patient for publication of this case report and any accompanying images. A copy of the written consent is available for review by the Editor-in-chief of this journal.

## Abbreviations

CT: Computed tomography.

## Competing interests

The authors declare that they have no competing interests.

## Authors’ contributions

AM drafted the manuscript. YK conceived the study and overall manuscript design. MM analyzed and interpreted the patient data. YT and SN participated in manuscript writing/revisions. TS performed the final editing of the manuscript. All authors read and approved the final manuscript.
